# Assessment of Diagnostic Techniques of Urinary Tuberculosis

**DOI:** 10.4084/MJHID.2013.034

**Published:** 2013-06-03

**Authors:** Khaled Ghaleb, Magdy Afifi, Mohamad El-Gohary

**Affiliations:** 1 Department of applied medical science, Faculty of Applied Medical Science, king Khalid University, Saudia Arabia, Bisha; 2 Department of Botany and Microbiology, Faculty of Science, Al-Azhar University, Assuit 71524, Egypt; 3 Department of Internal Medicine, Faculty of Medicine, Al-Azhar University, Assuit, Egypt

## Abstract

Early diagnosis of active tuberculosis remains an elusive challenge. In addition, one third of the world’s population is latently infected with Mycobacterium tuberculosis (Mtb) and up to 10% of infected individuals develop tuberculosis (TB) in their lifetime. In this investigation, the incidence of urinary tuberculosis among renal patients was studied. Three hundreds urine samples were processed for detection of Mtb by Ziehl-Neelsen (ZN) smear examination, Lowenstein Jensen (LJ) medium, radiometric BACTEC460 system as well as polymerase chain reaction (PCR) followed by DNA Enzyme Immunoassay (DEIA) test. Out of 300 urine samples, 2 were positive by both ZN smears and LJ medium with incidence rate of 0.66 %, 3 positive samples by BACTEC460 culture system with incidence of 1%. PCR assay gave more positive results than smear and culture examination (i.e. 8 positive samples with incidence rate of 2.6%). The specificities were 25% for both ZN smears and LJ medium, 37.5% for BACTEC460 culture system, and 100% for PCR test, while sensitivities of all assays were 100%. Thus PCR is a rapid and sensitive method for the early diagnosis of urinary tuberculosis.

## Introduction

Tuberculosis kills over 1.7 million people worldwide every year and nearly 40% of patients with active tuberculosis remain undiagnosed because of the poor sensitivity of the current, century old diagnostic method.^1^ The situation is further exacerbated with the increasing incidence of drug resistant TB.^2^ Early diagnosis of TB remains an elusive challenge, especially in individuals with disseminated TB and HIV co-infection.^3^

Early diagnosis plays a vital role in control of TB. Diagnosis of mycobacterial infections, however, remains an enigma. Although acid fast bacilli (AFB) microscopy and LJ culture remains the cornerstone of the diagnosis of TB, these traditional bacteriological methods are either slow or their sensitivity is quite low, especially with clinical samples that contain small number of organisms.^4^ This can affect treatment by either delaying it or causing inappropriate empiric therapy for TB to subjects without mycobacterial infections or with atypical mycobacteria.^5^

Urogenital tuberculosis (UGTB) is among the most common manifestations of extrapulmonary tuberculosis (EPTB) worldwide.^6^,^7^ Because of its insidious evolution and late onset of symptoms, the diagnosis and treatment are notoriously delayed, resulting in significant morbidity (e.g. end-stage renal failure, shrunken bladder, and testicular destruction)(). 8,9,10 UGTB is mainly caused by members of the *Mtb* complex. Nevertheless, several mycobacteria species other than tuberculosis are also associated with urogenital infections.^11^,^12^ Many efforts have been made to develop faster diagnostic assays for UGTB.^13^,^14^ However, the optimal treatment cannot be initiated on the basis of rapid tests alone, because they fail to assess mycobacterial viability or provide affordable phenotypic drug susceptibility testing. Similarly, fast diagnostic methods, such as matrix-assisted laser desorption ionization-time of flight-mass spectrometry or genotype, which are useful in tuberculosis lung infections, have the same drawbacks.^15^,^16^,^17^ Therefore, urine or tissue culture remains essential in the diagnosis of UGTB and its treatment.

UGTB is the second most common extrapulmonary presentation of tuberculosis, affecting 8–15% of the patients with pulmonary tuberculosis.^8^ From the lungs, the kidneys are affected through hematogenic dissemination, with subsequent involvement of the ureters and bladder through descending infection of the collecting system. The genital organs are involved through hematogenic (prostate and epydidimus) or retrograde canalicular dissemination.^8^,^18^ UGTB affects all age ranges, but predominates in men in their fourth or fifth decades. The diagnosis is often delayed, especially in developing countries, due to the evolution insidious with few and unspecific symptoms, along with a lack of awareness of physicians.^19^,^20^

Several studies have been done to detect *Mtb s* in urine and other clinical samples by, conventional ZN (Ziehl-Neelson) stained acid fast bacilli (AFB) microscopy and culture by LJ and radiometric BACTEC system^21^,^22^ as well as by amplifying different DNA sequences of *Mtb* by PCR test.^23^ In addition, the pathogen-specific biomarkers can be applied for the rapid and effective diagnosis of TB. It is likely that detection of a combination of biomarkers offers greater reliability of TB diagnosis, rather than detection of any single pathogen biomarker.^3^

The aim of this study was to compare different techniques for the diagnosis of renal tuberculosis, including Ziehle-Neelsen(ZN) smear, Lowenstein Jensen(LJ) medium, Bactec 460 radiometric culture system as well as PCR followed by DIA hybridization.

## Materials and Methods

Three hundred urine samples were collected from the outpatient department of the Azhar University’s hospital of the School of Medicine in Assiut, Egypt from January until June 2009. The samples were collected in the morning from the admitted patients from urology department in the hospital. The patients were divided into three groups (I, II, III), each group comprised 100 patients. Group 1 (control group), comprised apparently healthy patients, Group II, comprised a patient with chronic renal failure, and Group III, comprised patients with suspected infection (Table 1).

### Processing of specimens

Urine specimens were treated with NALC-NaOH method for the decontamination.^24^ Ziehl-Neelsen (ZN) staining method was done as described previously.^25^ Lowenstein-Jensen medium (Becton-Dinkinson Microbiology Systems, Cockeysille, MD, USA) was prepared.^26^ The processed specimens were inoculated into a Bactec 12 B vial.^24^ The BACTEC460 instrument (Becton Dickinson Diagnostic Instrument System, Sparks, MD, USA) was achieved, which was significantly superior to the conventional method.^22^

### Polymerase Chain Reaction (PCR). DNA extraction

The urine specimens were treated as previously mentioned for the decontamination and liquefaction to obtain the pellet for DNA extraction.^24^ The bacterial pellet was resuspended in 180 ul buffer ATL (supplied in QIAamp Tissue Kit, QIAGEN GmbH, Hilden, Germany). The extraction of TB DNA was performed according to QIAGEN kit protocol. The eluted DNA can be stored at −20°C until use in PCR.

### Primers and PCR assay

The PCR assay was performed with the primers pair p1(5’CCTGCGAGCGTAGCGTAGGCGG-‘3) p2(5’-CTCGTCCAGT CCAGCGCCGCTTCGG-‘3) located within IS6110 sequence repeated 10–12 times in the chromosome of *M. Tuberculosis*, these primers gives a 123 bp product and synthesized by Sorin BIomedica Diagnostics, Saluggia, VC, Italy which gives. Ten μl of the extract were subjected to PCR in a final volume of 100μl in 0.2ml microcentrifuge tubes containing 1X PCR buffer (10 mM Tris-HCl, pH 8.0,2.5 mM MgCl_2_, 50 mM KCl, gelatin 0.01%w/v), 0.2 mM (each) of the four deoxyribonucleoside triphosphates (dATP, dTTP, dGTP and dCTP), 2.5 U of taq polymerase were all supplied from STRATA-GENE reagent kit (Alameda, California, USA) and 50 pM each primer. The PCR conditions for IS6110 DNA amplification were an initial denaturation step of 94°C for 5 min, followed by 35 cycles of 94°C for 2min, 68°C for 2 min, 72° for 2min. and a final extension step at 72°C for 10min. The PCR procedure was accomplished with a thermocycler TC 9600 (Perkin-Elmer Cetus). Each experiment included positive and negative control tubes. The products of amplification were then analyzed by agarose gel electrophoresis followed by hybridization with DNA Enzyme Immunoassay (DEIA test).

### DNA Enzyme Immunoassay (DEIA test)

One hundred μl hybridization buffer were dispensed into each well, and 200μl controls and denatured samples into their respective wells. The strip was sealed with card board sealer, incubated at 45°C for 30 min and then washed. The anti-DS-DNA solution was prepared at the end of the first incubation and 100μl diluted Anti-DS. The DNA were dispensed into each well except for the blank, the strip was sealed and incubated for 1h. at room temperature, then washed. The enzyme tracer solution was prepared at the end of the incubation. Hundred μl diluted enzyme tracer solution were dispensed into all the wells except for the blank, the strip was sealed and incubated for 1 hr. at room temperature. The chromogen-substrate was prepared at the end of third incubation. At the end of incubation, substrate strip was washed. Hundred μl chromogen – substrate were dispensed into each well, incubated for 30min at room temperature, away from light. Two hundred μl of blocking reagent (GENE Kit DEIA, Sorin Biomedica Diagnostics, Saluggia, VC, Italy) were then dispensed into all the wells. The strip was read using ELISA reader photometer with 450–630 nm filter. The instrument was rested with blank, before reading the wells at 450nm. Absorbance values at least 0.170 greater than the mean negative control value indicated that hybridization had taken place.

### Statistical analysis

The incidences of all techniques were performed with the Statistical Package for Social Science **(**SPSS version 12.0**)** using the Z test and P value.

## Results

Characteristics of the studied patients are summarized in Table 1; 300 urine samples were provided into three groups (I, II, III) as previously mentioned.

The most common presenting symptoms in groups II and III patients with urinary TB were sterile pyuria (17% and 22 %), haematuria (2% and 4%) in group I and II, respectively. In addition, Dysuria (3%) and persistent dysuria (25%) are reported only in group II (Table 2 ).

Out of 300 urine specimens, 2 samples were detected by both ZN smear and LJ medium giving an incidence rate of 0.66 %, 3 samples were also found to be contaminated in BACTEC culture giving an incidence rate of 1%. Accordingly, PCR assay and non-isotopic hybridization probe test gave more positive results than smear and culture examinations (i.e. 8 samples were detected and gave an incidence rate of 2.6%). The results are given in Table 3.

The results show that ZN smear examination has a sensitivity of 25% and a specificity of 100%. For LJ media culture, sensitivity was 25% and specificity was 100%. BACTEC culture showed a sensitivity of 37.5% and a specificity of 100%. In comparison, PCR was found to have a much higher sensitivity of 100% and a specificity of 100% (Table 4).

The specificity, sensitivity and speed of PCR test in diagnosis of *M. tuberculosis* infection shown in this study should encourage the use of this technique in diagnosis of urinary TB. Acid-fast bacilli (AFB) smear were performed on all specimens. The reading of semiquantitative results had indicated the present number of bacteria (Table 5).

In this study, two positive specimens for *M. tuberculosis* were detected by both ZN smear and LJ medium out of 300 specimens, one positive specimen was found in group II and one positive specimen was found in group III (Table 3). The control specimens (group I) were completely negative by LJ medium.

Three positive specimens of *M. tuberculosis* were detected by Bactec culture system out of 300 urine specimens (1%). These positive samples were 1 in group II and 2 in group III. The control specimens (group I) were completely negative by Bactec460 culture (Table 3), while the sensitivity of BACTEC 460 system was 37.5 % and the specificity was 100% (Table 4).

**Figure 1:**
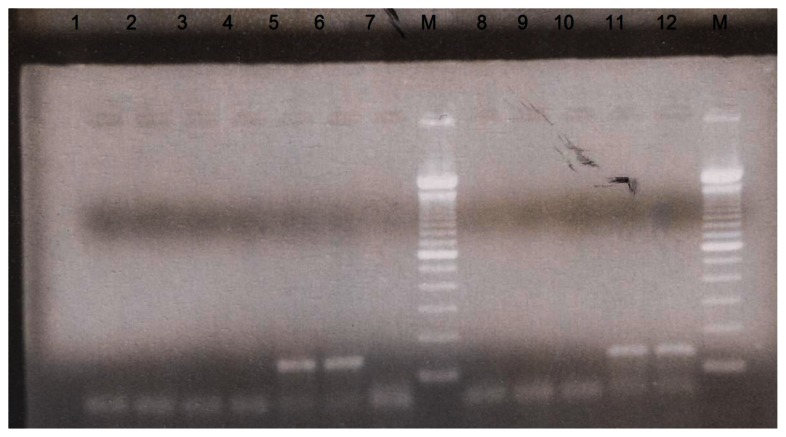
Agarose gel electrophoresis of amplified *Mycobacterium tuberculosis* DNA at 123 bp. Lanes 1, 2, 3, 4, 7, 8, 9, and 10 are negative. While 5, 6, 11, 12 are positive, M= 100 bp ladder.

The detection of *M. tuberculosis* DNA in urine specimens by PCR and non-isotopic hybridization was elevated for laboratory diagnosis of urinary tract *M. tuberculosis* infection. After amplifying DNA by PCR, the samples were detected by agarose gel electrophoresis to see the specific band for *M. tuberculosis*. Positive specimens were seen in agarose gel containing 123 bp band located within IS6110 sequence repeated 10–12 times in the chromosome of *M. tuberculosis*. The 100 bp ladder was used as depicted in Figure 1.

Negative specimens were shown by absence of 123 bp band. The results of gel electrophoresis of amplified product were 8 positive specimens for *M. tuberculosis*. Six of which had a clear band of 123 bp and other two had a weak or faint 123 bp band, these two specimens considered positive after confirmation using hybridization by non-isotopic hybridization to the specific probe. Hybridization to the specific probe was performed to all samples on the amplified products. Results of non-isotopic hybridization probe was performed and read at 450 nm, mean OD of the DEIA negative control was 0.05 A_450_ and positive control was 3.0 A_450_. Therefore, the OD cut-off value calculated in this study was 0.170 A_450_ and the samples were classified as negative at OD_450_< 0.170 or positive at OD_450_ ≥ 0.170. The eight positive specimens had OD in the range of 0.750–1.5 A_450_. The positive control used in PCR reaction gave OD value ≥2.0 and 0.450 A_450_.

To conclude, molecular diagnosis of tuberculosis by PCR has a great potential than ZN smear examination, LJ medium, and BACTEC radiometric culture, to improve the clinicians’ ability to diagnose urinary tuberculosis. This will ensure early treatment to patients and prevent further transmission of disease.

## Discussion

Early identification of tuberculosis in the clinical setting is of great importance in order for specific therapy to be swiftly initiated because TB is the leading cause of death worldwide attributable to a single infectious disease agent. UGTB is the second most common form of extrapulmonary tuberculosis in countries with a severe epidemic and the third most common form in regions with a low incidence of tuberculosis.^27^

Because of the insidious evolution and late onset of TB symptoms, the diagnosis and treatment are notoriously delayed, resulting in significant morbidity (e.g. end-stage renal failure, shrunken bladder, and testicular destruction).^8^,^9^,^10^

In our context, the most common presenting symptoms in patients in this study with UTB are sterile pyuria (17% and 22%), haematuria (2% and 4%) in group I and II, respectively. In addition, dysuria (3%) and persistent dysuria (25%) are reported only in group II.

Therefore, the diagnosis of UTB is often delayed not only because of its insidious evolution accompanied by few nonspecific symptoms, but also because of the long time required to confirm the diagnosis by classical conventional methods of cultivation,^28^ the only utilized in most developing countries.

Sterile pyuria (81%), pyuria (48%) and hematuria (18%) have been reported in patients with renal tuberculosis.^13^ Moreover, Narayan (29), found that the most common presenting symptoms in patients with UTB are irritative voiding symptoms and hematuria. Histopathology is characteristic but there could be problems to get a representative specimen, and non-specific features can sometimes complicate the diagnosis. Nevertheless, antigen-based serological tests have the potential of providing inexpensive and robust tools for diagnosis of TB under the conditions encountered commonly in developing countries such as Egypt.

However, immunological diagnosis is often inconclusive because antibodies and the delayed type hypersensitivity response persist for a long time after the subsidence of sub-clinical or clinical disease. Although a number of TB specific seroantigens have been identified in recent years, these vary in their sensitivities and specificities. Therefore, newer and reliable diagnostic methods are needed for early and precise detection of TB and efforts are ongoing in various laboratories to improve the performance of TB diagnostic tests either through improvements in conventional tools or by using newer diagnostic modalities that are based on nucleic acid amplification or antigen/antibody detection, etc. The salient achievements made towards improving the diagnosis of TB are summarized in this study.

In our investigation, the incidence of urinary tuberculosis among renal patients was studied. Three hundreds urine samples were processed for detection of *Mtb* by ZN smear examination, LJ medium culture, radiometric BACTEC460 culture as well as PCR and non-isotopic hybridization probe test.

The results show that ZN smear examination has a sensitivity of 25% and a specificity of 100%. To make such detection of UGTB faster, one could use media such as Middlebrook (solid or liquid) or Lowenstein-Jensen in the microcalorimetric vials, such as was proposed in other studies.^30^,^31^,^32^,^33^ Those media could then be inoculated with urine samples previously concentrated by centrifugation and decontaminated using the *N*-acetyl-L-cysteine-sodium hydroxide procedure to inactivate contaminants and avoid mixed bacterial growth.

The sensitivities of BACTEC culture method were found to be much higher compared to LJ i.e 37.5% and a specificity of 100% as well as the mean detection time for *Mtb* was 24.03 days by LJ medium culture, 12.89 days by BACTEC culture as reported (14,34,35).

Another noteworthy aspect of our study was in the diagnosis by PCR. Its results revealed 8 positive samples out of 300 specimens with incidence of 2.6%. Moreover, in comparison with the previous techniques, PCR was found to have the highest sensitivity of 100% and a specificity of 100%. This specificity, sensitivity and speed of PCR test followed by non-isotopic hybridization probe test in diagnosis of *M. tuberculosis* infection in this study, should encourage the use of this method in diagnosis of TB compared the performance of ZN smear, LJ media, and BACTAC 460 culture system in urine samples for diagnosis of TB as reported by others^34^,^23^,^21^ as well as Immunoassay test.^36^,^37^,^3^, ^38^ However, immunological diagnosis is often inconclusive because antibodies and the delayed type hypersensitivity response persist for a long time after the subsidence of sub-clinical or clinical disease. Although a number of TB specific seroantigens have been identified in recent years, these vary in their sensitivities and specificities.

With the use of PCR test and non-isotopic hybridization probe test, we are able to detect *M. tuberculosis* in 4 times than both ZN smear and LJ culture and 2.66 times than BACTAC460 culture system.

PCR test detected *M. tuberculosis* in less than one day, compared to average 24.03 days required for detection by conventional (LJ) and 12.89 days by radiometric BACTEC technique, as supported.^39^ Similar measurements using bactec culture and polymerase chain reaction methods have been performed for diagnosis of tuberculosis and found that a significant difference was seen in the sensitivities of 74.4% for PCR test, 33.79% for ZN smear examination, 48.9% for LJ culture and 55.8% for BACTEC culture (P<0.05). However, there was no significant difference (P>0.05) as far as specificity of different tests was concerned.^21^

PCR test was also shown to be reasonably sensitive in diagnosis of TB.^40^,^41^,^42^,^13^,^43^,^44^,^38^

Taken together, the present demonstration emphasized that among various diagnostic methods used for urinary tuberculosis, PCR, as a recent method, offers a promising new approach for rapid, safe, specific, and reproducible determination of *M. tuberculosis* infection.

## Figures and Tables

**Table 1:** Sampling criteria of the examined patients

Group No.	No. of specimens	Males		Females	
	
		No./100 (%)	Age (year) Mean ±SD	No./100(%)	Age (year)Mean±SD

I	100	34 (34)	46.5±6.5	66 (66)	42.5±8.0
II	100	83 (83)	44.0±10.0	17 (17)	39.5±16.5
III	100	75 (75)	37.5±7.5	25 (25)	37.0±9.0

I= normal urine patients without any urological complaint

II= Chronic renal failure patients

III= Suspected tuberculosis patients

**Table 2:** Clinical symptoms of groups II and III

Symptoms	Group II (%)	Group III ( %)

Sterile pyuria	17	22
Haematuria	2	4
Dysuria	-	3
Persistent dysuria	-	25

**Table 3:** Urinary TB among three different groups

Detection methods of *Mtb*	Group I	Group II	Group III	Total positivity n(%)
			
Methods	Positive specimens(%)	Positive specimens (%)	Positive specimens (%)	

Z.N. smear	0	1	1	2/300* (0.66%)
L.J.medium	0	1	1	2/300* (0.66%
Bactec 460 system	0	1	2	3/300 (1%)
PCR	0	2	6	8/300 (2.6%)

* significant value Z test (PCR versus ZN or LJ; p≤ 0.003)

**Table 4:** Sensitivity and Specificity of different methods.

Test performed	Sensitivity (%)	Specificity (%)

Z.N. smear	25	100
L.J. medium	25	100
BACTEC system	37.5	100
PCR test	100	100

**Table 5:** The detection **of semiquantitative** results to the **number** of Acid fast bacilli (AFB) cells of the examined urine specimens

Number of (AFB) cells / (OIF)	Semiquantitative results

0/300	Negative for AFB
1–2/300	± Repeat again
1–9/100	1^+^
1–9/10	2^++^
1–9/1	3^+++^
≥9/1	4^++++^

AFB =Acid fast bacilli

OIF= Oil Immersion Field.
